# Effects on health-related quality of life in patients treated with lurasidone for bipolar depression: results from two placebo controlled bipolar depression trials

**DOI:** 10.1186/s12888-016-0865-y

**Published:** 2016-05-23

**Authors:** Krithika Rajagopalan, Elizabeth Dansie Bacci, Daisy Ng-Mak, Kathy Wyrwich, Andrei Pikalov, Antony Loebel

**Affiliations:** Sunovion Pharmaceuticals Inc., Marlborough, MA USA; Evidera, Seattle, WA USA; Evidera, Bethesda, MD USA

**Keywords:** Quality of life enjoyment and satisfaction questionnaire, Patient functioning, Bipolar disorder, Depression, Drug therapy

## Abstract

**Background:**

Depressive symptoms associated with bipolar disorder negatively impact health-related quality of life (HRQoL). The efficacy of lurasidone in reducing depressive symptoms has been previously demonstrated. The objective of this study was to examine the direct and indirect effect (mediated through improvement in depression symptoms) of lurasidone in improving patient HRQoL.

**Methods:**

A secondary analysis of data was conducted of two 6-week, double-blind, placebo-controlled trials assessing the effect of lurasidone (lurasidone monotherapy [20–60 mg/day or 80–120 mg/day]; lurasidone adjunctive to lithium or valproate [20–120 mg/day]) in patients with bipolar depression. Patient HRQoL was measured using the Quality of Life Enjoyment and Satisfaction Questionnaire Short Form (Q-LES-Q SF). Depression symptoms were measured using the Montgomery-Asberg Depression Rating Scale (MADRS). Analysis of covariance (ANCOVA) was used to estimate the effect of lurasidone on improvement in the Q-LES-Q SF percentage maximum score from baseline to 6 weeks. Path analysis was used to evaluate the total effect (β_1_), as well as the indirect (β_2_*β_3_) and direct (β_4_) effect of lurasidone on Q-LES-Q SF change through improvements in MADRS.

**Results:**

A total of 340 and 485 patients from the monotherapy and adjunctive therapy, respectively, were included in the analysis. At 6-weeks, ANCOVA analyses demonstrated that lurasidone provided significant improvement in adjusted mean Q-LES-Q SF scores in comparison to placebo for monotherapy (22.9 and 22.7 vs. 15.2, both *p* < 0.01) and adjunctive therapy (23.1 vs. 17.9, *p* = 0.01). Path analyses indicated that lurasidone treatment predicted MADRS improvement (monotherapy: β_2_ = −0.44, *p* < 0.001; adjunctive therapy: β_2_ = −0.34, p = 0.003), which subsequently predicted improvement in Q-LES-Q SF (monotherapy: β_3_ = −0.73, *p* < 0.001; adjunctive therapy: β_3_ = −0.75, *p* < 0.001); however, the effect of lurasidone on improvement in Q-LES-Q SF was largely mediated by change in MADRS (monotherapy: β_4_ = 0.11, *p* = 0.13; adjunctive therapy: β_4_ = 0.02, *p* = 0.77).

**Conclusions:**

Lurasidone as a monotherapy and adjunctive to lithium or valproate is an effective treatment for improving HRQoL in patients with bipolar depression. However, improvement in HRQoL was not independent of improvement in depression, indicating that the effect of lurasidone on improving patient HRQoL may act through a reduction in depressive symptoms associated with bipolar disorder.

**Trial registration:**

Clinicaltrials.gov identifiers: NCT00868699 and NCT00868452 (both registered March 23, 2009)

## Background

Bipolar disorder is a chronic and debilitating mental illness, characterized by recurrent mood fluctuations between periods of depressive and manic symptoms, with an estimated lifetime prevalence of 1.0 % [1, 2] and is the 12^th^ leading cause of disability worldwide [3]. Frequent episodes of depression represent the most common symptomatic state in individuals with bipolar disorder [4, 5], where depressive episodes are associated with a greater negative impact on social, occupational, and general functioning than episodes of mania [4, 6–8]. Consequently, health-related quality of life (HRQoL) is also highly compromised in individuals with bipolar depression, where patients with bipolar depression report even greater worsening in their HRQoL and other indicators of functioning than patients with other mood disorders or mental illnesses [4, 9, 10].

Traditionally, clinical trials of treatment efficacy in patients with bipolar depression have focused on symptom remission (using the Diagnostic and Statistical Manual-IV-TR [DSM-IV-TR] or clinician-reported outcome assessments such as the Montgomery-Asberg Depression Rating Scale [MADRS] score ≤10 to signify remission status [11]). Increasingly, the ability of the patient to perform activities of daily living, engage and nurture social relationships, and function independently are being considered when evaluating treatment efficacy [4, 12, 13], as the burden of bipolar depression can persist even in patients considered to be in symptomatic remission [14, 15]. Given this, HRQoL assessments can provide an additional indicator of improvement in areas valued by the patient beyond symptomatic improvement [16].

Relatively few clinical trials have incorporated patient-reported outcome (PRO) measures to assess improvement in HRQoL in drug registration trials, although the number of studies incorporating these tools is increasing given the increasingly holistic view of improving patient’s lives in treating chronic illnesses such as bipolar disorder [4, 17]. A literature review conducted by Revicki et al. in 2005 suggested that HRQoL may improve as a result of treatment, although these studies were limited to a short follow-up duration and small sample size [18]. More recently, IsHak et al. [19] expanded on previous reviews to address if various forms of treatment (e.g., drug therapy, behavioral therapy) have an impact on bipolar disorder, demonstrating that several drug and psychotherapeutic treatment regimens were associated with improved HRQoL in this patient population.

Only two medications were approved for the treatment of acute bipolar depression as a monotherapy in the United States until 2013 (e.g., combination of olanzapine-fluoxetine and quetiapine) [20–22]. In two placebo-controlled clinical trials, the atypical antipsychotic lurasidone, approved by the US FDA as a monotherapy and adjunctive to lithium or valproate, was found to significantly improve symptoms of depression, HRQoL, and other PROs in patients with bipolar depression over a 6-week period [23, 24]. Thus, positive benefits in areas of life valued by the patient were achieved over the trial period. However, it is important to know if PRO improvements were a direct result of lurasidone treatment (a direct effect), or if this HRQoL improvement is achieved due to reductions in symptoms of depression (a completely-mediated indirect effect) or a combination of both, direct and indirect effects. With this information, clinicians and payors will have a much greater understanding of the total effects associated with lurasidone treatment on HRQoL. Thus, the aim of this study was to examine the direct and indirect effects (mediated through improvement in bipolar depression symptoms) of lurasidone in improving patient HRQoL.

## Methods

### Study design and data source

A secondary analysis of data from two randomized, 6-week, multicenter, double-blind, placebo-controlled, parallel-group studies conducted to assess the efficacy of lurasidone as a treatment for bipolar depression (NCT00868699 and NCT00868452) were used to assess the direct and indirect effects of lurasidone on HRQoL improvement. The study population in this analysis was the intention-to-treat (ITT) population. For monotherapy, the ITT population consisted of a total of 505 randomized patients receiving at least one dose of study medication at doses of 20–60 mg/day or 80–120 mg/day, with at least one post-baseline efficacy measurement for the MADRS. Following a washout period of at least 3 days, patients were randomly assigned in a 1:1:1 ratio via an interactive voice response system to receive 6 weeks of treatment with lurasidone, at flexible daily doses of either 20–60 mg or 80–120 mg, or 6 weeks of placebo. For adjunctive therapy, the ITT population consisted of a total of 672 randomized patients receiving at least one dose of study medication at a dose of 20–120 mg/day adjunctive to lithium or valproate, with at least one post-baseline efficacy measurement for the MADRS. Patients underwent stratified randomization, based on treatment with either lithium or valproate, to either adjunctive lurasidone 20–120 mg/day or placebo in a 1:1 ratio via an interactive voice response system. The dose of mood stabilizer was adjusted to maintain a serum level in the range of 0.6–1.2 mEq/liter for lithium or 50–125 for valproate throughout the study.

The patient population in both studies was adult patients (≥18 years old) with bipolar I disorder. All patients were experiencing a major depressive episode (DSM-IV-TR criteria, ≥ 4 weeks and <12 months in duration), with or without rapid cycling, without psychotic features, and with a history of at least one lifetime bipolar manic or mixed manic episode. Diagnosis was confirmed by the Mini-International Neuropsychiatric Interview [25] and the Bipolarity Index [26]. A MADRS [11] score ≥20 and a Young Mania Rating Scale (YMRS) score ≤12 were required at both screening and baseline. For both studies, efficacy assessments were obtained at baseline and weekly intervals. The primary efficacy endpoint was the mean change from baseline to week 6 in MADRS total score. At each study visit, a qualified site-based rater conducted the MADRS assessment; a second MADRS assessment was administered and scored by computer as part of a quality control process. Additional study design details, including a comprehensive list of patient entry criteria, as well as primary efficacy and safety analyses, have been published elsewhere [23, 24].

The study was approved by an institutional review board at each investigational site (from January 2009 to May 2010) and was conducted in accordance with the International Conference on Harmonisation Good Clinical Practices guidelines and with the ethical principles of the Declaration of Helsinki. Written informed consent was obtained from all participants prior to participation.

### Scales and assessments included in this analysis

#### Symptom assessment

##### Montgomery Asberg Depression Rating Scale (MADRS)

The patient’s primary bipolar depression symptoms were assessed using the MADRS, a clinician-administered depression rating scale developed from a larger scale to be sensitive to change [11]. The MADRS has 10 items, each scored on a 0–6 scale. A score of 0 indicates an absence of that symptom, and anchor point descriptors are given for scores of 0, 2, 4, and 6. Items assess many facets of depression, including sadness, tension, pessimism, suicidal thoughts, reduced sleep, and reduced appetite. A total score of 60 is possible, indicating greater depression severity.

#### Quality of life enjoyment and satisfaction questionnaire short form

The Q-LES-Q SF is a 16-item HRQoL measure of the degree of enjoyment and satisfaction experienced by patients in various areas of daily living [27]. The 16 items reduce to eight summary scales that reflect major areas of functioning: physical health, mood, leisure time activities, social relationships, general activities, work, household duties, and school/coursework. Each item is rated on a 5-point scale, ranging from 1 (very poor) to 5 (very good). The first 14 items are the same as the General Activities section of the regular Q-LES-Q form and are used to compute the raw score (Items 15 and 16 were not used in the present analyses). The sum of scores for items 1–14 can range from 14–70, and is expressed as a percentage (0–100) of the maximum total score that is achievable. Higher scores indicate better HRQoL.

The demographic and baseline characteristics of all participants were summarized using descriptive statistics (mean, standard deviation, range, frequencies for categorical variables). Descriptive statistics for the Q-LES-Q SF and the MADRS were summarized at baseline. All analyses were stratified by clinical trial (i.e., monotherapy and adjunctive therapy).

### Efficacy analyses

For the current analysis, mean change in Q-LES-Q SF percentage maximum scores (∆Q-LES-Q SF) from baseline to 6 weeks was used to assess the efficacy of treatment on HRQoL. Analysis of covariance analyses (ANCOVA) were conducted, with fixed effects for treatment group, pooled study center, and baseline score entered as covariates. Pairwise comparisons between means were performed using Scheffe's test adjusting for multiple comparisons. Model effect estimates, standard errors (SE), and *p*-values are presented for all analyses.

### Path analysis

Path analysis was conducted for monotherapy and adjunctive therapy trials (collapsing across dosage groups) to assess the relationship between treatment and ∆Q-LES-Q SF directly and through ∆MADRS, following the procedures described by Baron and Kenny [28]. The Baron and Kenny mediation model assesses the degree of the treatment effect upon a response variable in the presence of another variable (i.e., the mediating variable). This approach allows the examination of the degree of mediation (either as partial or complete mediation), through a series of four models. Statistically significant (*p* < 0.05) effects must be obtained in Models 1, 2, and 3 in order to test the full mediation model in Model 4. Following this approach, four models were estimated: (1) effect of treatment on ∆Q-LES-Q SF; (2) effect of treatment on ∆MADRS; (3) effect of ∆MADRS on ∆Q-LES-Q SF, independent of treatment; and (4) effect of treatment ∆Q-LES-Q SF, even when controlling for the effect of ∆MADRS. ∆MADRS would be considered a partial mediator on the relationship between lurasidone and ∆Q-LES-Q SF if statistically significant effects were achieved in all 4 models. Complete mediation would be indicated if the effect of treatment on ∆Q-LES-Q SF described in Model 4 was 0. Although the Baron and Kenny model was originally implemented using ordinary least squares regression, modern path analysis methods were utilized in the current study following the Baron and Kenny steps in order to accurately estimate the significance of the indirect effect.

Mplus statistical software version 7.0 [29] was used to conduct all analyses for Models 1–4. The total effect (β_1_) of the relationship between treatment and ∆Q-LES-Q SF was estimated in Model 1. The direct effect of treatment on the ∆Q-LES-Q SF controlling for ∆MADRS (β_4_), and the indirect effect, were estimated in Model 4. The indirect effect was calculated as the product of the relationship between treatment and ∆Q-LES-Q SF (β_2_), and the relationship between ∆MADRS to ∆Q-LES-Q SF (β_3_). Bias-corrected bootstrapping was used to estimate the standard errors for the estimation of the *p*-values to determine significance of each path, as well as the indirect effect. The proportion of the effect that was mediated was calculated as β_2_*β_3_/ β_1_, while the percentage of total variance explained by each path was reported using the standardized R^2^ values. The strength of the parameter estimates was interpreted using Kenny recommendations for effect size estimates of small (0.02), medium (0.15), and large (0.40) [30] for large samples with adequate power for detecting an effect. For the analysis, each of the models controlled for baseline score of the dependent variable (i.e., Q-LES-Q SF or MADRS). Parameter estimates with corresponding p-values were calculated for all four models for both trials.

Overall model fit was assessed using various global fit indices, where the following indices and fit values were used as criteria to assess acceptable model fit: chi-square test of overall model fit; Root Mean Square Error of Approximation (RMSEA) < 0.06 [31]; and Tucker Lewis Index (TLI) and the Comparative Fit Index (CFI) > 0.90 [32].

## Results

### Demographics and baseline characteristics

In the monotherapy trial, 818 patients were screened, of whom 505 (61.7 %) were randomly assigned to 6 weeks of treatment and 485 (96.0 %) were included in the ITT population. In the adjunctive therapy trial, a total of 672 patients were screened, of whom 348 (51.8 %) were randomly assigned to 6 weeks of treatment and 340 (97.7 %) were included in the ITT population. Study completion rates were similar for treatment and placebo groups for monotherapy (74 %, 73 %, and 75 %) and adjunctive therapy (78.1 % and 82.4 %) trials, respectively, in addition to baseline demographic and clinical characteristics for both studies (Table 1).

**Table 1:** Patient demographic characteristics at baseline

Variable	Monotherapy			Adjunctive therapy	
	Lurasidone 20–60 mg/day (*N* = 161)	Lurasidone 80–120 mg/day (*N* = 162)	Placebo (*N* = 162)	Lurasidone (*N* = 179)	Placebo (*N* = 161)
Age, mean (SD)	41.3 (12.3)	42.0 (12.4)	41.2 (12.4)	41.0 (11.5)	42.6 (11.8)
Male, n (%)	70 (43.5 %)	64 (39.5 %)	75 (46.3 %)	93 (52.0 %)	85 (52.8 %)
White, n (%)	107 (66.5 %)	106 (65.4 %)	107 (66.0 %)	108 (60.3 %)	102 (63.4 %)
Baseline Q-LES-Q SF Percentage Maximum Score, mean (SD)	51.3 (20.8)	52.1 (20.0)	45.5 (20.5)	55.9 (19.6)	50.4 (20.6)
Baseline MADRS Total Score, mean (SD)	30.3 (5.0)	30.6 (4.9)	30.5 (4.9)	30.5 (5.3)	30.7 (4.8)

### Efficacy results

Descriptive statistics for the Q-LES-Q SF at baseline and at 6 weeks are presented in Tables 2, 3, and 4, along with the corresponding change scores. For the monotherapy trial, change scores on the Q-LES-Q SF indicating improvement was significantly larger for both lurasidone 20–60 mg/day (mean change = 21.9) and 80–120 mg/day (mean change = 23.0) groups in comparison to placebo (mean change = 14.5). Similar change was demonstrated for adjunctive therapy for the lurasidone group (mean change = 23.3) in comparison to the placebo group (mean change = 17.9).

**Table 2:** Mean change for Q-LES-Q SF percentage maximum scores from baseline to 6 weeks: monotherapy (Lurasidone 20–60 mg/day and Lurasidone 80–120 mg/day)

	Lurasidone 20–60 mg/day			Lurasidone 80–120 mg/day		
	*N* Mean (SD)	*N* Mean Change (SD)	*P* value	*N* Mean (SD)	*N* Mean Change (SD)	*P* value
Q-LES-Q SF						
Baseline	157 33.8 (13.7)	--		160 33.5 (13.0)	--	
6-Weeks	123 55.9 (19.4)	120 21.9 (16.0)	<.0001	121 56.8 (18.1)	120 23.0 (17.7)	<.0001

All *p*-values for Q-LES-Q SF outcomes are based on *t*-tests comparing differences from baseline to 6 weeks within treatment group

**Table 3:** Mean change for Q-LES-Q SF percentage maximum scores from baseline to 6 weeks: monotherapy (combined lurasidone treatment groups and placebo)

	Combined treatment dose groups			Placebo		
	N Mean (SD)	N Mean Change (SD)	P value	N Mean (SD)	N Mean Change (SD)	*P* value
Q-LES-Q SF						
Baseline	317 33.7 (13.4)	--		160 34.2 (13.5)	--	
6-weeks	244 56.4 (18.7)	240 22.5 (16.9)	<.0001	125 48.7 (20.7)	124 14.5 (17.0)	<.0001

All *p*-values for Q-LES-Q SF outcomes are based on *t*-tests comparing differences from baseline to 6 weeks within treatment group

**Table 4:** Mean Change for Q-LES-Q SF percentage maximum scores from baseline to 6 weeks: adjunctive therapy (Lurasidone 20–120 mg/day and Placebo)

	Lurasidone 20–120 mg/day			Placebo		
	*N* Mean (SD)	*N* Mean Change (SD)	*P* value	*N* Mean (SD)	*N* Mean Change (SD)	*P* value
Q-LES-Q SF						
Baseline	177 36.1 (14.3)	--		159 35.7 (13.5)	--	
6-Weeks	142 58.4 (181)	141 23.3 (18.7)	<.0001	135 52.7 (19.9)	134 17.9 (20.2)	<.0001

All p-values for Q-LES-Q SF outcomes are based on *t*-tests comparing differences from baseline to 6 weeks within treatment group

In the ANCOVA analyses controlling for pooled study center and baseline Q-LES-Q SF percentage maximum score for the monotherapy trial, the least squares mean (SE) change from baseline to 6 weeks on the Q-LES-Q SF was significantly greater for lurasidone 20–60 mg/day group (22.9 [1.5], *p* < 0.001) and 80–120 mg/day group (22.7 [1.5], *p* < 0.01) in comparison to placebo (15.2 [1.5]). Similar findings were obtained in the adjunctive therapy trial for the lurasidone group in comparison to placebo (23.1 [1.5] versus 17.9 [1.6]; *p* <0.05).

### Direct and indirect effects of lurasidone on HRQoL

The total effect between lurasidone treatment and ∆Q-LES-Q SF in Model 1 was strong and statistically significant in both trials (Table 5). Relationships tested in Models 2 and 3 were also statistically significant (all *p* < 0.05). Thus, the full mediation model (Model 4) was analyzed and tested to determine if there was an independent effect of treatment on ∆Q-LES-Q SF after accounting for the variance explained by ∆MADRS.

**Table 5:** Path analysis of models 1, 2, and 3 for monotherapy and adjunctive therapy trials

Model	Monotherapy β (*p*-value)	Adjunctive therapy β (*p*-value)
Model 1	β_1_ = 0.45 (*p* < 0.001)	β_1_ = 0.28 (*p* < 0.05)
Model 2	β_2_ = −0.41 (*p* < 0.01)	β_2_ = −0.36 (*p* < 0.01)
Model 3	β_3_ = −0.77(*p* < 0.001)	β_3_ = −0.75 (*p* < 0.001)

**Fig. 1:**
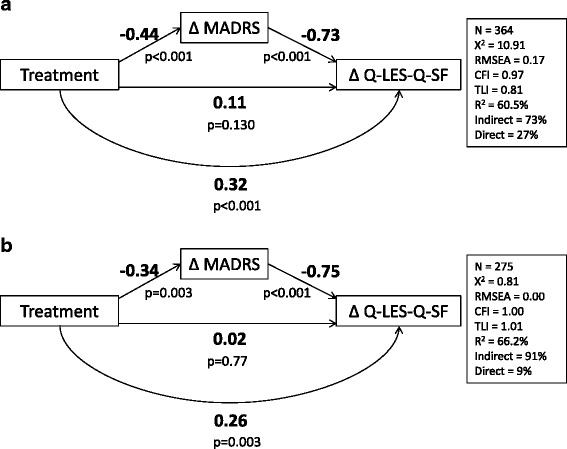
**a** Path analysis model 4: Monotherapy. **b** Path analysis model 4: adjunctive therapy

As shown in Fig. 1a and b (for monotherapy and adjunctive therapy, respectively), lurasidone treatment predicted improvement in MADRS (monotherapy = β_2_ = −0.44; adjunctive therapy: β_2_ = −0.34), which subsequently predicted improvement in HRQoL (monotherapy: β_3_ = −0.73; adjunctive therapy: β_3_ = −0.75). However, the direct effect of treatment on ∆Q-LES-Q SF after accounting for ΔMADRS did not remain statistically significant in either study, as the direct effect estimate was considered small (monotherapy: β_4_ = 0.11, *p* = 0.13) to near 0 (adjunctive therapy: β_4_ = 0.02, *p* = 0.77). This finding is reflected in the indirect effects, estimated as 0.32 in the monotherapy trial (*p* < 0.001) and 0.26 (*p* < 0.01) in the adjunctive therapy trial. The percentage of variance accounted for by the indirect effect through ∆MADRS was 73 % and 91 %, respectively. For both studies, the full mediation models with direct and indirect effects explained the majority of the variability in ∆Q-LES-Q SF (monotherapy: R^2^ = 60.5 %; adjunctive therapy: R^2^ = 66.2 %). The findings from this analysis provide evidence for partial mediation in the monotherapy trial and almost complete mediation in the adjunctive therapy trial.

## Discussion

In this post-hoc analysis of two 6-week, randomized, placebo-controlled clinical trials conducted to assess the efficacy of lurasidone on HRQoL in patients with bipolar depression, lurasidone performed significantly better in comparison to placebo in improving patient-reported HRQoL. In analyses of lurasidone as a monotherapy and adjunctive therapy to lithium or valproate, similar adjusted change in the Q-LES-Q SF was evidenced for all treatment groups receiving lurasidone. The overall findings provide evidence that lurasidone performs similarly as a monotherapy and as an adjunctive therapy, at a range of dosage levels, in improving self-reported HRQoL in patients with bipolar depression.

While morbidity and mortality is high in patients with bipolar depression and HRQoL is often greatly compromised, improvement in HRQoL in clinical treatment trials for bipolar depression is often understudied. However, the relatively few studies that have been published provide support for the assertion that treatment can improve HRQoL in patients with bipolar depression. Using the Medical Outcomes Study 36-Item Short-Form Health Survey (SF-36) as a measure of HRQoL in an 8-week randomized, controlled trial of olanzapine or olanzapine-fluoxetine combination, Shi and colleagues [33] found that patients receiving either olanzapine or olanzapine-fluoxetine reported significantly greater improvement on the mental and physical component scores of the SF-36. More recently, Endicott and colleagues [34] conducted a secondary analysis of two 8-week randomized, controlled clinical trials of quetiapine. Using a mixed model for repeated measures analysis, they found that patients taking 300 and 600 mg/day significantly improved on the Q-LES-Q SF with adjusted least squares mean (SE) change scores of 10.89 (0.59) and 12.14 (0.62), respectively, by Week 8. These findings parallel earlier work where Q-LES-Q SF scores were significantly improved in patients taking quetiapine in comparison to placebo at Week 4 and Week 8 [35]. The present study demonstrated that in controlled analyses, lurasidone was even more effective in improving HRQoL as measured by the Q-LES-Q SF, where mean (SD) change scores were 22.5 (16.9) and 23.3 (18.7) in the monotherapy and adjunctive therapy studies, respectively.

The HRQoL improvement in patients receiving lurasidone parallel the reduction in MADRS depression scores in this sample [23, 24]. Specifically, both as a monotherapy and an adjunctive therapy, lurasidone treatment had a significantly greater effect in reducing depression over 6 weeks in comparison to placebo as measured by the MADRS and the Clinical Global Impressions-Bipolar scale. Path analysis revealed that MADRS total score improvement was a partial (monotherapy) and almost-complete mediator (adjunctive therapy) on the relationship between treatment and improvement in the Q-LES-Q SF. Thus, improvement in HRQoL in lurasidone-treated patients largely occurred through improvements in depression symptoms. This finding of partial (rather than full) mediation in the monotherapy trial could be due to larger mean Q-LES-Q SF improvement resulting from lurasidone administered as a monotherapy in comparison to an adjunctive therapy. These findings are supported by previous investigations comparing the effects of monotherapy and combination therapy on HRQoL using the investigator-rated global assessment of functioning (GAF) in patients with bipolar disorder [36, 37]. However, future research assessing the incremental benefit of monotherapy over adjunctive therapy on HRQoL improvement is needed in patients with bipolar depression.

This knowledge of both the direct and indirect effects of lurasidone treatment on improvement in HRQoL provides a more holistic view of the complex effect of treatment on the magnitude and level of improvement in how a patient feels or functions. Whereas the primary goal of treatment for patients with bipolar depression is often the alleviation of depression symptoms, improved HRQoL is often critical to patients and their clinicians [4], and could potentially shape long-term recovery. For example, improved HRQoL has been associated with greater adherence to treatment [38], which has been shown to be a great barrier to effective long-term treatment in patients with bipolar depression [39].

The results of the present study are supported by previous investigations that have demonstrated that a strong relationship exists between a depressed clinical state and reduced HRQoL in patients with bipolar disorder [34, 40–42]. For example, using data from the Systematic Treatment Enhancement Program for Bipolar Disorder (STEP-BD) multicenter trial, researchers sought to determine the unique contribution of clinical states (e.g., depression, mania/hypomania, mixed) in relation to HRQoL. Bipolar patients with depression had significantly lower HRQoL scores in comparison to recovered, recovering, and patients with mania/hypomania, controlling for sociodemographic and health characteristics. Similar conclusions were drawn by Saarni et al. [43] and Vojta [41], concluding that in bipolar patients, depressive symptoms are the strongest predictors of reduced HRQoL. Also using the Q-LES-Q SF and MADRS to assess HRQoL and depression, respectively, Endicott [34] found that improvement in HRQoL across an 8-week period was most strongly correlated with improvements in MADRS scores. In the current study, we expanded on these analyses using path analysis, finding that not only is change in these constructs correlated across time, but that change in MADRS is predictive of change in Q-LES-Q SF, thus providing an assessment of the mediating effect of depression on the treatment-HRQoL relationship.

Several study limitations should be noted. The study duration was only 6 weeks, and a longitudinal study is needed to determine if patients maintain the demonstrated improvement or continue to improve over a longer period of time. However, a 6-week trial length is a standard for acute depression clinical trials [44, 45]. In addition, HRQoL was assessed based on patient-reported data, and not direct observation of patient behavior or functioning, and the assessment was conducted with only one assessment of HRQoL. The findings from the current study could be different if an alternative PRO of HRQoL was selected. Finally, we used path analysis in a limited sample to determine if depression mediated the treatment-HRQoL relationship. A replication of these analyses in a larger population is needed to confirm our findings. Although mediation analysis allows for the interpretation of causal relationships from correlational data, additional research using a causal study design is necessary to validate these findings.

## Conclusions

In summary, patients with bipolar depression have greatly impaired HRQoL. Lurasidone as a monotherapy (20–60 mg/day and 80–120 mg/day) and adjunctive to lithium or valproate (20–120 mg/day) was efficacious in improving HRQoL in this study. Change in HRQoL is largely mediated by improvement in depression symptoms. These findings underscore the significance of treatment effectiveness in reduction of depressive symptoms, which in turn result in improvement of HRQoL in patients with bipolar depression.

## Abbreviations

ANCOVA: Analysis of covariance
CFI: Comparative Fit Index
DSM-IV-TR: Diagnostic and Statistical Manual-IV-TR
GAF: Global assessment of functioning
HRQoL: Health-related quality of life
ITT: Intention-to-treat
∆MADRS: Mean change in MADRS total score
MADRS: Montgomery-Asberg Depression Rating Scale
PRO: Patient-reported outcome
∆Q-LES-Q SF: Mean change in Q-LES-Q SF percentage maximum scores
Q-LES-Q SF: Quality of life enjoyment and satisfaction questionnaire short form
RMSEA: Root mean square error of approximation
SE: Standard errors
STEP-BD: Systematic treatment enhancement program for bipolar disorder
TLI: Tucker lewis index
YMRS: Young mania rating scale
